# Identification of adjuvants for clinical trials performed with *Plasmodium falciparum* AMA1 in rabbits

**DOI:** 10.1186/s12865-019-0307-y

**Published:** 2019-07-30

**Authors:** Sumera Younis, Bart W. Faber, Clemens H.M. Kocken, Edmond J. Remarque

**Affiliations:** Biomedical Primate Research Centre, Department of Parasitology, Rijswijk, The Netherlands

**Keywords:** AMA1, *Plasmodium falciparum*, Adjuvants, SE, SE-GLA, Liposomes, Liposomes-GLA, CoVaccine HT™, ImSaVac-P, Rabbits

## Abstract

**Background:**

In this study, seven adjuvants were compared for use with *Plasmodium falciparum* DiCo-Apical Membrane Antigen 1 (*Pf*-DiCo-AMA1), with the aim to identify an ideal adjuvant which yields high antibody titres and potentially broadens the responses in clinical trials. The following adjuvant formulations were evaluated: SE, SE-GLA, Liposomes, Liposomes-GLA, CoVaccine HT™, ImSaVac-P and ImSaVac-P o/w. The study was performed in rabbits, which were immunized with FVO-AMA1 in combination with one of the seven adjuvants. Antibody levels (humoral responses) and functional activity of the antibodies induced against malaria vaccine candidate AMA1 were evaluated. Thus, in this study the ideal adjuvant is expected to induce high functional antibody levels, a long-lived response, and a broad cross-strain activity.

**Results:**

AMA1 formulated in all adjuvants was immunogenic. However, the magnitude of the immune responses differed between the seven adjuvants. The highest IgG levels were observed for the CoVaccine HT™ group, this was statistically significant for all four AMA1 variants versus all other adjuvant groups. No differences were observed in the breadth of the humoral response, i.e., increased recognition of AMA1 variants. Also, Growth Inhibition Activity (GIA) for both *Plasmodium falciparum* strains (FCR3 – homologous to FVO AMA1 protein and NF54 – heterologous to FVO AMA1 protein) were significantly higher in the CoVaccine HT™ group as compared to the other adjuvant groups.

**Conclusions:**

In brief, all seven vaccine – adjuvant formulations were immunogenic. The magnitude of the immune responses differed between the seven adjuvants. No statistically significant differences were observed in the breadth of the humoral response, nor in longevity of the response. Nevertheless, AMA1 formulated in CoVaccine HT™ appeared as the best adjuvant for use in clinical trials.

**Electronic supplementary material:**

The online version of this article (10.1186/s12865-019-0307-y) contains supplementary material, which is available to authorized users.

## Background

Malaria has been a burden on humans throughout recorded history, currently especially in developing countries. It is one of the world’s most common and serious tropical diseases, caused by parasites of the phylum Apicomplexa belonging to the genus *Plasmodium*. Malaria caused by *Plasmodium falciparum* is responsible for 196–263 million infections per year and an annual death toll of more than 445.000 people especially in Sub Saharan countries [1].

Young children and pregnant women are the most vulnerable to malaria infections. The need for an effective vaccine is high, because of the high mortality and drug resistance of malaria parasites against existing drugs. Previous research has shown that *P. falciparum* (*Pf*) merozoite membrane protein Apical Membrane Antigen 1 (AMA1) is a promising asexual blood stage vaccine candidate against malaria, reviewed by Remarque et al, 2008 [2].

Full length *Pf*AMA1 (83 kDa) is initially located in the micronemes of the merozoites. At the time of merozoite release it is processed to a 66 kDa protein and translocated to the merozoite surface, where it is involved in the complex sequence of red blood cell invasion [3–5]. It is known that AMA1 is polymorphic [6]. The polymorphism is generated due to single amino acid substitutions [7]. Alignment of 2372 *Pf*AMA1 sequences showed that around 140 (22%) of the 622 amino acid residues can vary between alleles and that linkages between polymorphic residues occur. In the past studies with rodent malaria parasite *Plasmodium chabaudi* showed that polymorphism in AMA1 negatively affected vaccine outcomes [8]. Also rabbit immunization studies have shown that antibodies to *Pf*AMA1 obtained from one malaria strain inhibit the growth of other strains to a much lesser degree [9, 10], suggesting that *Pf*AMA1 polymorphism may diminish the efficacy of *Pf*AMA1 single variant-based vaccines, and that the most effective AMA1 vaccines should induce immune responses to all variants. Hence, to cover the variation between different alleles three Diversity Covering (DiCo) sequences were produced, previously described by Remarque et al, 2008, these sequences incorporate 97% of the amino acid variability [11].

As protein-based subunit vaccines are less immunogenic when administered alone, adjuvants, carrier proteins or Virus-Like Particles (VLP) are required to induce high-titered, long-lasting immune responses. The use of an adjuvant modulates the magnitude and type of immune response to a vaccine. In the past numerous studies were performed with AMA1 formulated in adjuvants [12, 13]. For almost 80 years, aluminium salts, like Alhydrogel®, have been the only adjuvant used in human vaccines and was used as a gold standard to compare other adjuvants. Although Alhydrogel® is generally well-tolerated in humans, studies in humans have shown that Alhydrogel® is a relatively weak adjuvant for antibody induction to malaria antigens [2, 14]. In another attempt to obtain high levels of functional AMA1 antibodies, Mullen et al, 2006 tested AMA1 in combination with Alhydrogel® alone and in combination with Alhydrogel® combined with a Toll-Like Receptor 9 (TLR9) receptor agonist, CPG7909 [14]. Co-administration of AMA1 formulated in Alhydrogel® with CPG7909 resulted in higher antibody titres compared to Alhydrogel® alone [14]. In mice, addition of a TLR9 agonist also skewed towards a Th1 response, with an 8–10-fold increase of IgG2a antibody levels.

Furthermore, clinical studies showed that a single allele 3D7-AMA1 vaccine formulated in AS02A and AS01B was immunogenic, with functional antibody responses against homologous strain and demonstrable IFN-γ responses [15]. AS02A is an oil-in-water (o/w) emulsion mixed with two immunostimulants monophosphoryl lipid A (MPL) and saponin derivative QS-21. AS01B is a manufacturing proprietary liposomal formulation with the same proportions of MPL and QS-21 found in AS02A [16]. The AMA1 based malaria vaccine FMP2.1/AS02A produced high and lasting antibody responses, although growth rates were reduced by only 17%, which is insufficient to effectively reduce parasite multiplication. In a Phase IIb study, however, 64.3% efficacy was observed against homologous AMA1 strain, with only 17% overall efficacy underscoring the importance of the breadth of the response [17].

Previous research on AMA1 and adjuvants shows the need of a comparison study to identify adjuvants to be used for *Pf-*DiCo-AMA1 in clinical trials [18]. AMA1 immunity is assumed to be antibody mediated [2, 19], therefore the ideal adjuvant to be used in clinical trials is expected to induce high antibody levels, a long-lived response, and broad cross-strain activity. The aim of this study is to identify adjuvant formulations for *Pf-*DiCo-AMA1 for use in clinical trials and evaluate the immunogenicity and ultimately the efficacy of the vaccine – adjuvant formulations. The single FVO allele of AMA1 will be used instead of the generated DiCo antigen, as use of the single allele AMA1 will give the opportunity to observe the potential broadening of the response besides the magnitude of the response with the different formulations.

In our comparison study the FVO AMA1 vaccine candidate will be used in combination with the following seven adjuvant formulations: Stable o/w Emulsion (SE), SE-Glucopyranosyl Lipid A (GLA), Liposomes, Liposomes-GLA, CoVaccine HT™, ImSaVac-P and ImSaVac-P (o/w). The choice of adjuvant was dependent on availability of GMP material. SE and Liposomes are two vehicles that can be complemented with TLR4 agonists, such as GLA or water soluble LpxL (ImSaVac-P). Previous studies have shown that synthetic TLR4 adjuvants can enhance the magnitude and quality of protective immunity induced by influenza vaccines [20]. Moreover, addition of a TLR4 agonist to an adjuvant skews the response towards a Th1 response yielding higher IgG2a and IgG2c levels in mice. SE and Liposomes were used alone as well as formulated with GLA, whereas the water soluble TLR4 agonist ImSaVac-P was used alone or in an o/w emulsion. CoVaccine HT™, a proprietary adjuvant formulation (BTG International Ltd., United Kingdom), was also included in this comparative study. CoVaccine HT™ is an o/w emulsion-based vaccine adjuvant, which consists of synthetic sucrose fatty acid sulphate esters (SFASES) immobilized inside the oily droplets of the submicron squalane in water emulsion [21]. The rationale for the use of CoVaccine HT™ is: (i) Rhesus monkeys immunized with *Pk*AMA1 formulated in CoVaccine HT™ were able to control parasitaemia in a *P. knowlesi* challenge model [22]. (ii) The DiCo-AMA1 formulated in CoVaccine HT™ induced high Growth Inhibition Assay (GIA) titres in Rhesus monkeys. (iii) Additionally, it was also shown that CoVaccine HT™ yielded high antibody responses in rabbits [23].

In brief, the aim of this study was to identify adjuvant formulations yielding high antibody titres and potentially broadening the responses for clinical trials with *Pf-*DiCo-AMA1. The ideal adjuvant was expected to induce high functional antibody levels, a long-lived response, and a broad cross-strain activity. Previous rabbit immunization studies presented distinguishable results with homologous and heterologous AMA1 alleles [13]. Therefore, this study was conducted in rabbits in order to evaluate humoral responses and functionality of the humoral responses by GIA.

## Methods

### Adjuvants and vaccine preparation

Vials containing 62.5 mcg of lyophilized clinical grade FVO-AMA1 [10, 24] were reconstituted with Saline (0.9%) to 12 mcg/mL. 250 μL of the AMA1 solution was then mixed with 250 μL of one of the seven adjuvants: SE (IDRI-EM064), SE-GLA (IDRI-EM062), Liposomes (IDRI-LS119), Liposomes-GLA (IDRI-LS118) were supplied by the Infectious Disease Research Institute (IDRI Seattle, WA), CoVaccine HT™ was supplied by Protherics BTG (London UK) [21, 22], ImSaVac-P and ImSaVac-P o/w were supplied by ImSaVac Technologies B.V. (Utrecht, The Netherlands) [25].

Rabbits received 3.0 mcg AMA1 in 500 μL intramuscularly (i.m.). All formulations were stored at 4 °C until use. Vaccines were used within 4 h of preparation, see Table 1. Based on the supplier’s advice, GLA was used in rabbits at 50 mcg per dose. This is 5- to 10-fold higher than the human dose [26], as rabbits are considered hypo-responsive to GLA. The total amount of SFASES per dose (10 mg) was based on safety data obtained in rhesus macaque vaccine studies [22, 23] and published data on SFASES in pigs [27]. ImSaVac-P dose (10 mcg) was also based on the supplier’s advice.

**Table 1:** Injection volumes and dose of immune enhancers for rabbits

Group	Volume	Dose
SE	500 μL (i.m.)	n.a.
SE-GLA	500 μL (i.m.)	50 mcg
Liposomes	500 μL (i.m.)	n.a.
Liposomes-GLA	500 μL (i.m.)	50 mcg
CoVaccine HT™	500 μL (i.m.)	10 mg
ImSaVac-P	500 μL (i.m.)	10 mcg
ImSaVac-P o/w	500 μL (i.m.)	10 mcg

*n.a.* not applicable

Final formulations are 500 μL consisting of equal amounts of AMA1 solution and Adjuvant. Amounts in the table are for the final formulation as injected

Dose is the amount of GLA, SFASES (CoVaccine HT™) or ImSaVac-P in one injection of the specified formulation

### Animal immunization

All animal work was performed under the guidelines of BioGenes GmbH, Germany, which adopt protocols fully complying with European animal welfare regulations, regulating ethical issues on laboratory animal treatment. Immunization work at BioGenes GmbH was under approval from NIH/OLAW (ID number #A5755–01).

Immunization studies were carried out in groups of twelve adult female rabbits. The rabbits were immunized three times at 4-weeks intervals (day 0, 28 and 56). Eight animals per group were exsanguinated at week 10 (day 70) and four animals were further sampled at 4-weeks intervals and exsanguinated at week 20 (day 140) (Table 2). The animals were euthanized in accordance with the Directive 2010/63/EU, as euthanasia practice a captive bolt followed by exsanguination was used.

**Table 2:** Immunization scheme

Day	
0	1st immunization
28	2nd immunization
56	3rd immunization
70	8 animals exsanguinated
140	4 animals exsanguinated

A low dose of AMA1 (3.0 mcg) was selected for rabbits in this study in order to be able to better distinguish adjuvant effects. The AMA1 used for immunisation was the GMP product used in a previous clinical trial [24, 28]. On day 0 pre-vaccination samples were collected.

### ELISA

To evaluate humoral responses Enzyme-Linked ImmunoSorbent Assay (ELISA) was performed on serum samples of rabbits in 96-well flat bottom Microlon titre plates (Greiner, Alphen a/d Rijn, The Netherlands). Plates were coated overnight with 1 μg/mL (100 μL/well) of the relevant AMA1 antigen (FVO, HB3, CAMP or 3D7) at 4 °C. The antigens used were either GMP-produced (FVO) or lab produced using the similar methodology as for the GMP product [24]. The *P. pastoris*-expressed AMA1 from 3D7, HB3 and CAMP used in the ELISA’s differ by 26, 20 and 17 amino acid positions in the ectodomains (aa 25–545) from the FVO vaccine allele (Table 3) [11]. After blocking with 200 μL/well of 3% BSA (Sigma, Zwijndrecht, The Netherlands) in PBS-T samples were loaded on the plates.

**Table 3:** Number of amino acid variants between *P. pastoris*-expressed AMA1 and 3D7, HB3 and CAMP

Vaccine Antigen	Amino acid variants with *P. pastoris*-expressed AMA1				
	Total	Prodomain	Domain I	Domain II	Domain III
3D7	**26**	2	17	5	2
HB3	**20**	2	11	4	3
CAMP	**17**	3	9	3	2

Numbers in bold represent amino acid differences for AMA1 ectodomain

Day 0, day 70 and day 140 rabbit sera samples were loaded on the plates and incubated for 2 h at RT. Day 0 samples were tested at a 1:100 and 1:500 dilutions for total IgG. Day 70 and day 140 samples were tested at 1:5000 in a three-fold serial dilution over five wells. BG98 Rabbit IgG was used as a standard starting at 600 ng/mL total IgG in a 3-fold dilution series over 7 wells, this standard was generated by pooling antibodies of 98 rabbits which were immunized with seven-antigen mixtures in CoVaccine HT™ [13]. After sample incubation, plates were incubated with 100 μL/well of 1:1250 diluted goat anti-rabbit IgG conjugated to alkaline phosphatase (Thermo Fisher Scientific, Etten-Leur, The Netherlands). ELISA development was with 100 μL/well p-nitrophenyl phosphate (pNPP; Fluka, Poole, UK) for 30 min. The optical density (OD) was read at 405 nm using the BioRad platereader (model iMark – microplate reader).

The Four Parameters Logistic Fit was used to convert the ODs to arbitrary units (AUs) - ADAMSEL, www.malariaresearch.eu. One AU yields an OD of 1 over background, So, the amount of AU of a sample is the reciprocal dilution at which an OD of 1 over background is achieved. On every plate a standard curve was included [29].

### Parasites

The NF54 and FCR3 strains of *P. falciparum* were maintained in culture medium, RPMI 1640 (Gibco, Invitrogen, Breda, The Netherlands) supplemented with 10% heat inactivated O+ human serum and 15 mcg/mL Gentamycin (Invitrogen, Breda, The Netherlands) at 5% hematocrit. Culture medium was changed daily and when required the culture was diluted with human red blood cells (O+) to maintain a parasitemia at approximately 1.5%. Cultures were incubated at 37 °C in 5% O_2_, 5% CO_2_, and 90% N_2_ atmosphere. Parasitemia was determined by microscopy of Giemsa (Merck, Schiphol-Rijk, The Netherlands) stained blood smears. The *Pf*AMA1 antigen expressed by all parasite strains was confirmed by PCR and restriction fragment length analysis. Parasite cultures were mycoplasma-free and synchronized twice with 0.3 M Alanine, 10 mM Hepes pH 7.5 before use in assays.

### Immuno fluorescence assay

Synchronous cultures of NF54 and FCR3 mainly at schizont stage were used to prepare IFA slides. Culture was washed trice with RPMI, spinning for 5 min at 2000 rpm. After centrifugation Fetal Calf Serum (FCS - Gibco, Invitrogen, Breda, The Netherlands) was added in 1:1 ratio to the pellet. Thin smears were prepared on multitest 12-well slides (MP Biomedicals, Eindhoven, The Netherlands). Slides were stored in slide boxes placed in a plastic bag containing desiccant - silica gel (Sigma, Zwijndrecht, The Netherlands) at − 80 °C until use.

At room temperature, thawed slides were fixed with cold methanol for 10–60 s. 15 μL of day 70 obtained sera (primary antibody) at different dilutions, starting at 1:1000 in 2-fold, was added to each slide well and incubated for 1 h at RT. After five PBS (Gibco, Invitrogen, Breda, The Netherlands) washes, slides were incubated again for 1 h in a moist box with secondary antibody Goat-anti-Rabbit-FITC (Thermo Fisher Scientific, Etten-Leur, The Netherlands) diluted 100x in PBS containing 1% FCS. Then slides were washed again five times. Nuclei were stained with DAPI at 1:5000 in antifade (Sigma, Zwijdrecht, The Netherlands). As a positive control sera obtained from BG98 rabbits was used [13]. The IFA titre is expressed as an end-point titre, i.e. the highest dilution at which a positive reaction was observed.

### Antibody purification

Antibodies were purified from day 70 and day 140 rabbit sera on protein G column (Sigma, St. Louis, MO). Protein G matrix was washed with two bed volumes of Binding Buffer (Thermo Fisher Scientific, Etten-Leur, The Netherlands). Diluted and filtered serum samples at a ratio of 1:2 were passed over the matrix once. After sample application columns were washed with two bed volumes of Binding Buffer, followed by 35–40 bed volumes of PBS. IgG antibodies were then eluted using 4–6 bed volumes Elution Buffer (Thermo Fisher Scientific, Etten-Leur, The Netherlands). Elution fractions were pH adjusted using 1/5 volume of Binding Buffer and subsequently filter sterilised through a 2-μm filter. Fractions were exchanged into RPMI 1640 using ethanol sterilised Amicon Ultra-15 concentrators (MilliPore, Amsterdam, The Netherlands). Next fractions containing antibodies were applied to the concentrators and centrifuged for 30–45 min at 3000 rpm. IgG concentrations were determined using a NanoDrop ND-1000 spectrophotometer (Nanodrop Technologies, Wilmington, DE, USA). The concentration of the purified and exchanged antibodies was adjusted to 12 mg/mL and antibodies were kept at − 20 °C until use.

### Growth inhibition assays

To study the functionality of the humoral responses, Protein G-purified IgG fractions of rabbit sera were tested in parasite GIA [30–32]. All IgGs were tested in triplicate on FCR3 (one amino acid difference in the pro-domain from the FVO strain, with *AMA1* GenBank accession no. M34553) and NF54 (parent strain of the 3D7 clone with *AMA1* GenBank accession no. U65407) parasite strains at 2-fold serial dilutions over four wells from 6 mg/mL to 0.75 mg/mL in 96-well half area cell culture plates (Greiner, Alphen a/d Rijn, The Netherlands). In all growth inhibition assays late trophozoite/early schizont stages at a parasitemia of 0.2–0.4 and 2% final hematocrit were used. The final culture volume per well was 50 μL and parasites were incubated in presence of purified antibodies for 42–46 h. After 42–46 h, cultures were resuspended, and 50 μL was transferred into plates containing 200 μL ice cold PBS. The plates were then centrifuged for 10 min at 1300*x* g at 4 °C, the supernatant was discarded and plates were frozen until parasite lactate dehydrogenase (pLDH) analysis. Parasite growth was assessed by measuring pLDH levels [31, 32]. After 30 min of development plates were read at 655 nm. Parasite growth inhibition was expressed as;

$$ \% inhibition=100-\frac{\left({A}_{655}\; Sample-{A}_{655}\; RBC\right)}{\left({A}_{655}\; SZ-{A}_{655}\; RBC\right)}\times 100 $$

Where *A*_*655*_*Sample* is the OD_655_ for any test sample well, *A*_*655*_*SZ* is the average OD_655_ of schizont control wells included on each plate and *A*_*655*_*RBC* is the average OD_655_ of RBC control wells. The data is presented as the arithmetic mean % inhibition from each sample triplicate [33].

### Statistical analysis

All statistical analyses were performed with the R language and environment for statistical computing version 3.4.3 (R Foundation for Statistical Computing, Vienna, Austria. ISBN 3–900051–07-0, URL http://www.R-project.org). Antibody levels were log-transformed to obtain a normal distribution. Between group antibody level comparisons were performed by Analysis of Variance (ANOVA). Between group differences are expressed as ratios with 95% confidence intervals. *P* values are adjusted for multiple comparisons using Tukey’s honest significant difference test (Tukey’s HSD test). A value of *p* < 0.05 was considered significant.

## Results

### Humoral responses in rabbits

#### IgG levels

**Fig. 1:**
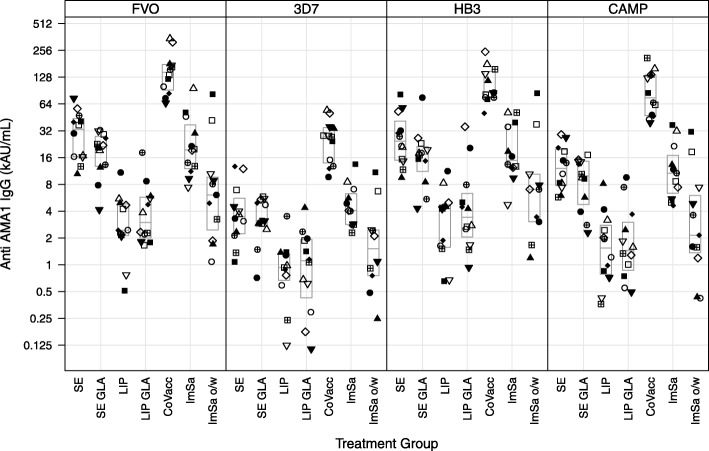
Total IgG levels in rabbit sera obtained on day 70. FVO AMA1 antigen (homologous) and 3D7, HB3, CAMP AMA1 antigen (heterologous). Same symbol within each treatment group refers to the same animal in all graphs. Boxes represent median and quartile ranges

IgG levels measured in rabbit sera two weeks after the third immunization are shown in Fig. 1. The highest IgG levels were observed for CoVaccine HT™; this was statistically significant for all four AMA1 ELISA coating antigens versus all other adjuvant groups. CoVaccine HT™ showed five times higher IgG titres compared to the SE and SE-GLA groups. IgG levels were intermediate in the SE, SE-GLA and ImSaVac-P groups, and lowest in the Liposomes, Liposomes-GLA and ImSaVac-P o/w groups. IgG levels to heterologous AMA proteins were lower and reflected the antigenic distance between the proteins, with CAMP being closest, HB3 intermediate, and 3D7 most different from the homologous FVO antigen.

The breadth of the IgG response was not different for the adjuvants tested. Because the use of AU/mL for IgG quantification, no direct IgG level comparisons can be made between the different alleles. Statistics on comparison between treatment groups for total IgG in day 70 sera for all four AMA1 alleles are shown in Additional file 1. Statistics on comparison between treatment groups for total IgG in day 140 sera for all four AMA1 alleles are shown in Additional file 2. Between group differences are expressed as ratios with 95% confidence intervals and *P* values are adjusted for multiple comparisons using Tukey’s honest significant difference test (Tukey’s HSD test). The fold-difference between the adjuvant comparisons revealed no significant differences (as judged by comparing the point estimates and confidence intervals), across the various alleles tested indicating similar breadth for all adjuvants (Additional files 1 and 2). The ratios of total IgG in day 140/day 70 sera to the homologous FVO AMA1 antigen and 3 heterologous variants (*N* = 4) in rabbits are shown in Additional file 3. The comparison reveals that between 6 and 19% of the day 70 titre to the FVO antigen is retained after 10 weeks.

**Fig. 2:**
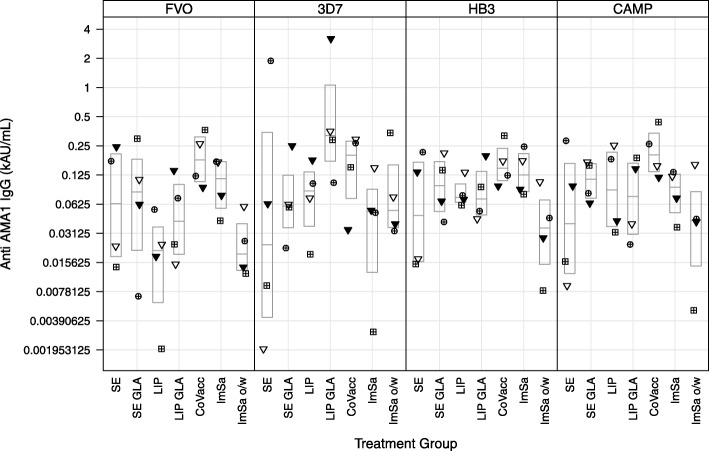
Ratio of total IgG levels in day 140/day 70 rabbit sera. FVO AMA1 antigen (homologous) and 3D7, HB3, CAMP AMA1 antigen (heterologous). Same symbol within each treatment group refers to the same animal in all graphs. Boxes represent median and quartile ranges

Geometric mean titres of levels of total IgG in day 70 and day 140 sera to all four AMA1 alleles are shown in Tables 4 and 5, respectively. Clearly, titres of rabbits in the CoVaccine HT™ groups are highest amongst other adjuvant groups. IgG levels decreased in sera obtained at day 140 compared to day 70. This decrease in AMA1-specific IgG levels was statistically significant. The decrease between day 70 and day 140 levels was least pronounced in the CoVaccine HT™ group (Fig. 2). Geometric mean titre ratios of total IgG in day 140/day 70 to all four alleles are shown in Additional file 4. The geometric means of total IgG in day 140/day 70 confirm that decrease in IgG levels obtained at day 140 compared to day 70 was least pronounced in the CoVaccine HT™ group compared to the other adjuvant groups.

**Table 4:** Geometric mean titres of levels of total IgG in day 70 sera to the homologous FVO AMA1 antigen and 3 heterologous (3D7, HB3, and CAMP) variants (*N* = 12) rabbits

Group	FVO	3D7	HB3	CAMP
Stable o/w Emulsion (SE)	27769 (18492 to 41701)	3624 (2230 to 5890)	25493 (16707 to 38898)	12243 (8530 to 17572)
SE-Glucopyranosyl Lipid A (GLA)	17526 (11819 to 25989)	3130 (2114 to 4633)	15695 (9761 to 25239)	8239 (5288 to 12837)
Liposomes	2683 (1570 to 4586)	805 (440 to 1473)	2894 (1619 to 5175)	1486 (822 to 2687)
Liposomes-GLA	3667 (2283 to 5892)	859 (427 to 1725)	4114 (2099 to 8064)	1716 (942 to 3128)
CoVaccine HT™	140707 (100939 to 196143)	24151 (16906 to 34501)	102988 (77022 to 137709)	82696 (57257 to 119436)
ImSaVac-P	21088 (12988 to 34240)	4574 (3166 to 6609)	18190 (11392 to 29044)	11197 (6908 to 18149)
ImSaVac-P o/w	6490 (2675 to 15749)	1554 (678 to 3559)	6961 (2714 to 17853)	2814 (1109 to 7142)

**Table 5:** Geometric mean titres of levels of total IgG in day 140 sera to the homologous FVO AMA1 antigen and 3 heterologous (3D7, HB3, and CAMP) variants (*N* = 4) rabbits

Group	FVO	3D7	HB3	CAMP
Stable o/w Emulsion (SE)	240 (14 to 4064)	22 (1 to 519)	74 (4 to 1471)	49 (2 to 1057)
SE-Glucopyranosyl Lipid A (GLA)	124 (5 to 3303)	40 (2 to 828)	56 (3 to 1111)	63 (4 to 976)
Liposomes	2 (> 0 to 23)	1 (> 0 to 8)	3 (1 to 13)	4 (> 0 to 42)
Liposomes-GLA	12 (2 to 70)	18 (3 to 108)	7 (2 to 23)	5 (2 to 15)
CoVaccine HT™	3397 (884 to 13049)	858 (54 to 13566)	1320 (218 to 7983)	1764 (280 to 11099)
ImSaVac-P	272 (163 to 453)	60 (10 to 376)	112 (42 to 301)	109 (29 to 408)
ImSaVac-P o/w	36 (6 to 222)	22 (3 to 180)	17 (1 to 342)	18 (1 to 553)

### Antibody detection

**Fig. 3:**
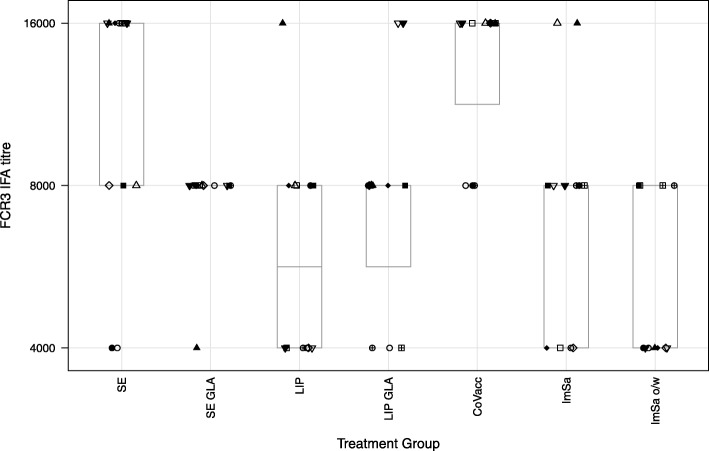
IFA titres in day 70 rabbit sera on FCR3 AMA1 *P. falciparum* strain (homologous). Same symbol within each treatment group refers to the same animal in all graphs. Boxes represent median and quartile ranges

All rabbit serum samples were assessed by Immunofluorescence Assay *P. falciparum* strain FCR3. The FCR3 strain expresses an AMA1 that is homologous to the immunizing FVO protein (one pro-sequence amino acid difference from FVO AMA1). All sera produced detectable IFA signals on both *P. falciparum* strains, confirming the ELISA results that immunizations were successful and suggesting that sera raised against AMA1 formulated in all seven adjuvants can react with the native AMA1 antigen on *P. falciparum* parasites. IFA results for FCR3 strain are shown in Fig. 3.

### Functionality of humoral responses

#### Functional antibody levels

**Fig. 4:**
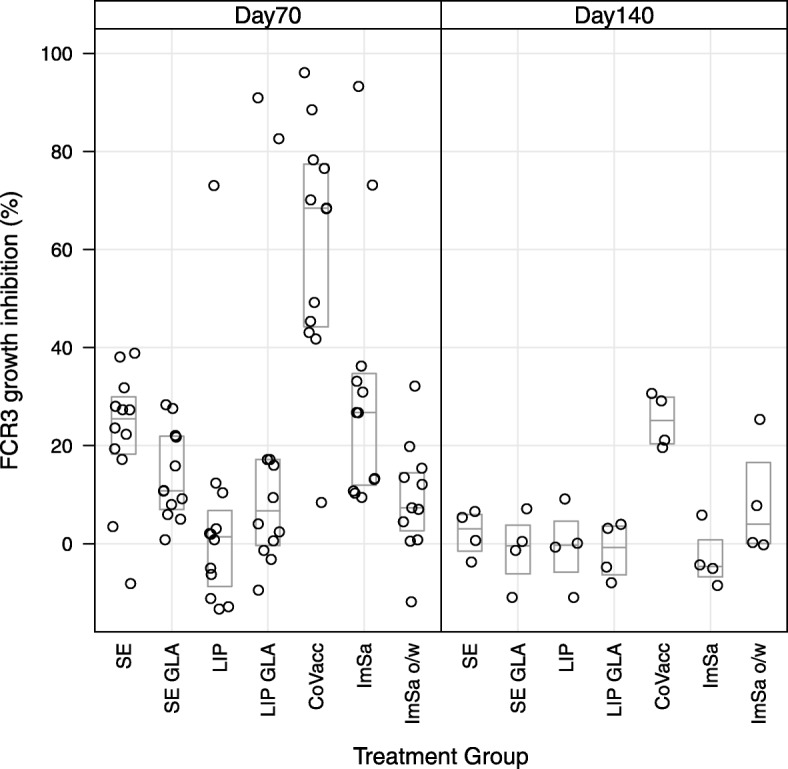
Antibody growth-inhibitory activity obtained with rabbit sera against FCR3 (homologous AMA1 strain). Day 70 left panel and Day 140 right panel. Boxes represent median and quartile ranges

**Fig. 5:**
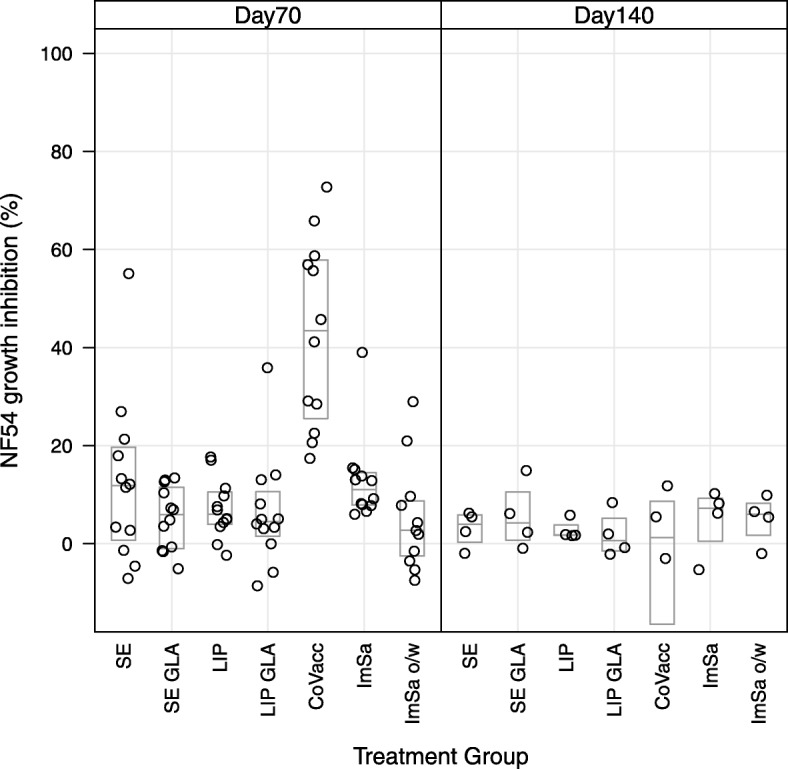
Antibody growth-inhibitory activity obtained with rabbit sera against NF54 (heterelogous AMA1 strain). Day 70 left panel and Day 140 right panel. Boxes represent median and quartile ranges

Growth inhibition assays (GIA) were performed again with the two laboratory strains expressing AMA1 variants, FCR3 and NF54. The FCR3 strain expresses an AMA1 that is homologous to the immunized FVO protein, whereas NF54 expresses an AMA1 that differs 26 amino acids in the ectodomain. Percentage growth inhibition obtained using IgG’s tested in 2-fold from 6 mg/mL to 0.75 mg/mL collected on day 70 and day 140 are shown in Fig. 4 for FCR3 and Fig. 5 for NF54 strain. Percentage growth inhibition for the FCR3 strain was significantly higher in the CoVaccine HT™ group at both time points as compared to all other adjuvant groups. A similar picture was observed for the NF54 strain on day 70, where again percentage growth inhibition was significantly higher in the CoVaccine HT™ group as compared to all other groups.

At day 140 the percentage growth inhibition decreased as compared to day 70 levels for all adjuvants for the homologous FCR3 strain. A similar trend was observed for the heterologous NF54 strain, except for the SE-GLA group where a slight increase (neglectable) was observed (from 5.3 (1.2 to 9.4) at day 70 to 5.6 (− 5.3 to 16.5) at day 140). Nevertheless, growth inhibitions below 20% are considered to be not above background variation [19]. CoVaccine HT™ is the only adjuvant with high detectable growth inhibition (61.2% (45.7 to 48.2)) at day 70 against FCR3 strain. Heterologous growth inhibition against NF54 was 42.9% (30.8 to 55.0) at day 70. However, at day 140 the growth inhibition is decreased to 25.1% (16.3 to 34.0) and − 3.9% (− 33.1 to 25.3) against FCR3 and NF54, respectively.

Raw data of the percentage growth inhibition at 6 mg/mL rabbit IgG are shown in Table 6. Statistics on comparison between treatment groups for GIA in day 70 and 140 at 6 mg/mL rabbit IgG for the two laboratory strains (FCR3 and NF54) are shown in Additional file 5. Between group differences are expressed as delta’s with 95% confidence intervals. *P* values are adjusted for multiple comparisons using Tukey’s honest significant difference test (Tukey’s HSD test). Statistics on comparison shows that growth inhibition activity obtained within the CoVaccine HT™ group was significantly higher compared to the other adjuvant groups for both laboratory strains.

**Table 6:** Percentage growth inhibition in day 70 and day 140 at 6 mg/mL rabbit IgG for the two laboratory strains (FCR3 and NF54)

Group	FCR3 day 70	FCR3 day 140	NF54 day 70	NF54 day 140
Stable o/w Emulsion (SE)	22.5 (13.9 to 31.0)	0.8 (−10.5 to 12.1)	12.6 (1.8 to 23.4)	3.1 (−2.8 to 9.0)
SE-Glucopyranosyl Lipid A (GLA)	14.1 (7.7 to 20.5)	−1.2 (− 13.0 to 10.7)	5.3 (1.2 to 9.4)	5.6 (−5.3 to 16.5)
Liposomes	4.6 (−10.1 to 19.3)	−0.6 (− 13.7 to 12.5)	7.2 (3.3 to 11.0)	2.8 (− 0.5 to 6.0)
Liposomes-GLA	18.9 (−2.0 to 39.8)	−3.2 (− 17.4 to 11.0)	6.4 (− 0.8 to 13.7)	1.9 (− 5.6 to 9.3)
CoVaccine HT™	61.2 (45.7 to 76.7)	25.1 (16.3 to 34.0)	42.9 (30.8 to 55.0)	−3.9 (− 33.1 to 25.3)
ImSaVac-P	31.4 (14.7 to 48.2)	−3.0 (−12.8 to 6.8)	12.9 (7.3 to 18.6)	4.9 (−6.2 to 15.9)
ImSaVac-P o/w	9.2 (1.5 to 17.0)	8.3 (−10.7 to 27.3)	5.3 (−2.2 to 12.8)	5.0 (−3.0 to 13.0)

## Discussion

The need for an effective vaccine against malaria is growing fast since the malaria parasite has acquired resistance to drugs commonly used to treat malaria (like chloroquine) [34] and resistance to Artemisinin-based combination therapy (ACT) [35]. Researchers all over the world put many efforts in this area of research. So far RTS,S is the only potential subunit vaccine that targets the pre-erythrocytic stage of the disease. RTS,S provided a modest protection against clinical and severe malaria in young infants in a Phase III trial [36]. Nevertheless, development of a combination vaccine covering pre-erythrocytic and blood-stages of the malaria parasite is expected to be more effective [37]. Therefore, investigations regarding a blood-stage vaccine candidate are necessary. To increase the immune response and development of antigen-specific immunity, vaccine candidates like DiCo-AMA1 are complemented with adjuvants. The study described in this paper is an evaluation of the immunogenicity and efficacy of the AMA1-FVO vaccine candidate formulated in seven different adjuvant formulations. AMA1-FVO was used instead of DiCo-AMA1, as use of the single allele AMA1 gives the opportunity to observe potential broadening of the response besides the magnitude of the response with the different formulations. Results of this study are a helpful asset to identify adjuvant formulations for *Pf-*DiCo-AMA1 to use in the clinical trials. To select adjuvants for the clinical trials, three selection criteria were selected: peak response (induction of high functional antibody levels), a long-lived response, and broad cross-strain activity.

Using these criteria, CoVaccine HT™ performed best in our rabbit immunization study. It yielded superior antibody responses, both in terms of function (GIA) as well as IgG levels, to all four AMA1 variants tested. A correlation between GIA levels and AMA1-specific IgG titers has been demonstrated by several investigators [19, 38–40]. No differences in breadth of the antibody response were observed when comparing the seven adjuvants, confirming a previous finding by Kusi et al*,* 2010 [13]. Antibody levels decreased markedly 84 days following the third vaccination and no differences in decrease rate were observed, except for the CoVaccine HT™ group where the decrease between day 70 and day 140 levels was least pronounced. Nevertheless, comparison of adjuvants should be based on day 70 titres in order to retain functional activity following the last vaccination. Antibody levels against FVO-AMA1 decreased considerably between days 70 and 140, with only 8–20% of day 70 IgG levels retained which is also reflected in the GIA titres. The decrease may be a result of the low dose used for immunisation, the vaccination regime, and or the use of rabbits. Data obtained in the clinical trial of FVO-AMA1, show that about 8–20% of IgG levels are retained between days 84 and 365 in humans [2], which is alike the decrease observed over 10 weeks in the current study. The data from the clinical trial illustrated that none of the adjuvants tested were able to induce sufficient longevous antibody levels, high enough to maintain GIA activity, suggesting that booster doses may be required [2]. Furthermore, for clinical trials DiCo-AMA1 vaccine antigen will be used and in a recently conducted clinical study by Sirima et al. DiCo-AMA1 induced high and long-lasting IgG responses [26]. Heterologous GIA activity is lost at day 140, which reflects the lower starting levels, further underscoring the need for booster vaccinations. Our findings are not specific for FVO-AMA1. Booster vaccinations were also required in studies performed with RTS,S. Efficacy was enhanced by the administration of a booster dose in children (age 5–17 months) and in young infants (age 6–12) [41].

In rabbits the three adjuvants SE, SE-GLA, and ImSaVac-P were almost equal in the amount of IgG and GIA induced. Similar data were found when total IgG antibody levels were evaluated in mice: CoVaccine HT™ induced the highest-ranking total IgG titers, while and SE, SE-GLA, Liposomes-GLA, ImSaVac-P all yielded lower and similar total IgG levels (unpublished data, Younis et al.). The amount of SFASE in CoVaccine HT™ (viz. 10 mg) is 3 logs higher than GLA, but no adverse events were observed, besides temporary elevations in neutrophil count and concomitant drops in free serum iron which resolved within two weeks when CoVaccine HT™ was tested in rhesus monkeys [19, 23]. Hence, the amount of SFASE is safe to use. Furthermore, a recent paper on SFASE biosimilars [42] reveals that CFASES (carbohydrate fatty acid sulphate esters) like the one used in CoVaccine HT™ are potent adjuvant components, but also induce transient temperature rises in rabbits. Moreover, Hilgers et al. also show that a mono-sulphorylated CFASE does not activate TLR4 [42], but acts through a different mechanism.

The choice of the runner-up adjuvant is not as clear-cut, as there are a few adjuvants performing almost equally. Another criterion in adjuvant selection was the Good Manufacturing Practice (GMP) status of the adjuvants. Currently, SE and SE-GLA are more advanced than the Liposomal or ImSaVac-P formulations and, therefore, it seems logical to restrict the choice of the second adjuvant to SE or SE-GLA. In rabbits, the amount of total IgG appears similar for SE and SE-GLA. The GIA titers in rabbits appear slightly, but not significantly, lower for SE-GLA than for SE.

## Conclusions

In this study, we have shown that the promising AMA1 vaccine candidate formulated in seven different adjuvants, SE, SE-GLA, Liposomes, Liposomes-GLA, CoVaccine HT™, ImSaVac-P and ImSaVac-P o/w resulted in pronounced, different immunogenicity profiles (humoral responses). All seven vaccines – adjuvant formulations were immunogenic. However, the magnitude of the immune responses differed between the seven adjuvants.

The highest IgG levels were observed for the CoVaccine HT™ group, this was statistically significant for all four AMA1 variants versus all other adjuvant groups. No differences were observed in the breadth of the humoral response, i.e., increased recognition of AMA1 variants. Also, Growth Inhibition Activity (GIA) for both *Plasmodium falciparum* strains (FCR3 – homologous to FVO AMA1 protein and NF54 – heterologous to FVO AMA1 protein) were significantly higher in the CoVaccine HT™ group as compared to the other adjuvant groups.

Concluding, AMA1 formulated in CoVaccine HT™ appeared as the best adjuvant in our study for use in clinical trials.

## Additional files

Additional file 1: Comparison between treatment groups for total IgG in day 70 sera for 4 AMA1 alleles in rabbits. Between group differences are expressed as ratios with 95% confidence intervals. *P* values are adjusted for multiple comparisons using Tukey’s honest significant difference test (Tukey’s HSD test). (XLSX 15 kb)

Additional file 2: Comparison between treatment groups for total IgG in day 140 sera for 4 AMA1 alleles in rabbits. Between group differences are expressed as ratios with 95% confidence intervals. P values are adjusted for multiple comparisons using Tukey’s honest significant difference test (Tukey’s HSD test). (XLSX 12 kb)

Additional file 3: Ratio of total IgG in day 140/day 70 sera to the homologous FVO AMA1 antigen and 3 heterologous variants (*N* = 4) in rabbits. (XLSX 13 kb)

Additional file 4: Comparison between treatment groups for day 70/140 titre ratios for 4 AMA1 alleles. (XLSX 9 kb)

Additional file 5: Comparison between treatment groups for GIA in day 70 and 140 at 6 mg/ mL Rabbit IgG for 2 laboratory strains. Between group differences are expressed as delta’s with 95% confidence intervals. P values are adjusted for multiple comparisons using Tukey’s honest significant difference test (Tukey’s HSD test). The FCR3 strain expresses an AMA1 homologous to the immunizing antigen, whereas NF54 strain expresses an AMA1 that differs by 26 amino acids in the ectodomain. (XLSX 12 kb)

## Data Availability

All data will be made available upon request to all interested researchers.

## Abbreviations

Aa: Amino acids
AMA1: Apical Membrane Antigen 1
ANOVA: Analysis of variance
AU: Arbitrary unit
CFASES: Carbohydrate fatty acid sulphate esters
DiCo: Diversity Covering
ELISA: Enzyme-Linked ImmunoSorbent Assay
GIA: Growth inhibition assay
GLA: Glucopyranosyl Lipid A
GMP: Good Manufacturing Practice
i.m.: Intramuscularly
IgG: Immunoglobulin-G
OD: Optical density
Pf: *Plasmodium falciparum*
pLDH: Parasite lactate dehydrogenase
pNPP: p-nitrophenyl phosphate
RBC: Red blood cell
RT: Room temperature
SE: Stable emulsion
SFASES: Synthetic sucrose fatty acid sulphate esters
SZ: Schizont
TLR: Toll-Like Receptor
VLP: Virus-Like Particles

## References

[1] Organization WH (2017). World malaria report 2017.

[2] Remarque Edmond J., Faber Bart W., Kocken Clemens H.M., Thomas Alan W. (2008). Apical membrane antigen 1: a malaria vaccine candidate in review. Trends in Parasitology.

[3] Dutta S, Haynes JD, Barbosa A, Ware LA, Snavely JD, Moch JK, Thomas AW, Lanar DE (2005). Mode of action of invasion-inhibitory antibodies directed against apical membrane antigen 1 of Plasmodium falciparum. Infect Immun.

[4] Polley SD, Mwangi T, Kocken CH, Thomas AW, Dutta S, Lanar DE, Remarque E, Ross A, Williams TN, Mwambingu G (2004). Human antibodies to recombinant protein constructs of Plasmodium falciparum apical membrane antigen 1 (AMA1) and their associations with protection from malaria. Vaccine.

[5] Weiss GE, Gilson PR, Taechalertpaisarn T, Tham WH, de Jong NW, Harvey KL, Fowkes FJ, Barlow PN, Rayner JC, Wright GJ (2015). Revealing the sequence and resulting cellular morphology of receptor-ligand interactions during Plasmodium falciparum invasion of erythrocytes. PLoS Pathog.

[6] Thomas AW, Waters AP, Carr D (1990). Analysis of variation in PF83, an erythrocytic merozoite vaccine candidate antigen of Plasmodium falciparum. Mol Biochem Parasitol.

[7] Chesne-Seck ML, Pizarro JC, Vulliez-Le Normand B, Collins CR, Blackman MJ, Faber BW, Remarque EJ, Kocken CH, Thomas AW, Bentley GA (2005). Structural comparison of apical membrane antigen 1 orthologues and paralogues in apicomplexan parasites. Mol Biochem Parasitol.

[8] Crewther PE, Matthew ML, Flegg RH, Anders RF (1996). Protective immune responses to apical membrane antigen 1 of Plasmodium chabaudi involve recognition of strain-specific epitopes. Infect Immun.

[9] Healer J, Murphy V, Hodder AN, Masciantonio R, Gemmill AW, Anders RF, Cowman AF, Batchelor A (2004). Allelic polymorphisms in apical membrane antigen-1 are responsible for evasion of antibody-mediated inhibition in Plasmodium falciparum. Mol Microbiol.

[10] Kocken CH, Withers-Martinez C, Dubbeld MA, Van Der Wel A, Hackett F, Valderrama A, Blackman MJ, Thomas AW (2002). High-level expression of the malaria blood-stage vaccine candidate Plasmodium falciparum apical membrane antigen 1 and induction of antibodies that inhibit erythrocyte invasion. Infect Immun.

[11] Remarque EJ, Faber BW, Kocken CH, Thomas AW (2008). A diversity-covering approach to immunization with Plasmodium falciparum apical membrane antigen 1 induces broader allelic recognition and growth inhibition responses in rabbits. Infect Immun.

[12] Aucouturier J, Dupuis L, Deville S, Ascarateil S, Ganne V (2002). Montanide ISA 720 and 51: a new generation of water in oil emulsions as adjuvants for human vaccines. Expert Rev Vaccines.

[13] Kusi KA, Faber BW, Riasat V, Thomas AW, Kocken CH, Remarque EJ (2010). Generation of humoral immune responses to multi-allele PfAMA1 vaccines; effect of adjuvant and number of component alleles on the breadth of response. PLoS One.

[14] Mullen GE, Giersing BK, Ajose-Popoola O, Davis HL, Kothe C, Zhou H, Aebig J, Dobrescu G, Saul A, Long CA (2006). Enhancement of functional antibody responses to AMA1-C1/Alhydrogel, a Plasmodium falciparum malaria vaccine, with CpG oligodeoxynucleotide. Vaccine.

[15] Spring MD, Cummings JF, Ockenhouse CF, Dutta S, Reidler R, Angov E, Bergmann-Leitner E, Stewart VA, Bittner S, Juompan L (2009). Phase 1/2a study of the malaria vaccine candidate apical membrane antigen-1 (AMA-1) administered in adjuvant system AS01B or AS02A. PLoS One.

[16] Brando C, Ware LA, Freyberger H, Kathcart A, Barbosa A, Cayphas S, Demoitie MA, Mettens P, Heppner DG, Lanar DE (2007). Murine immune responses to liver-stage antigen 1 protein FMP011, a malaria vaccine candidate, delivered with adjuvant AS01B or AS02A. Infect Immun.

[17] Thera MA, Doumbo OK, Coulibaly D, Laurens MB, Ouattara A, Kone AK, Guindo AB, Traore K, Traore I, Kouriba B (2011). A field trial to assess a blood-stage malaria vaccine. N Engl J Med.

[18] Mata E, Salvador A, Igartua M, Hernandez RM, Pedraz JL (2013). Malaria vaccine adjuvants: latest update and challenges in preclinical and clinical research. Biomed Res Int.

[19] Remarque EJ, Roestenberg M, Younis S, Walraven V, van der Werff N, Faber BW, Leroy O, Sauerwein R, Kocken CH, Thomas AW (2012). Humoral immune responses to a single allele PfAMA1 vaccine in healthy malaria-naive adults. PLoS One.

[20] Coler RN, Baldwin SL, Shaverdian N, Bertholet S, Reed SJ, Raman VS, Lu X, DeVos J, Hancock K, Katz JM (2010). A synthetic adjuvant to enhance and expand immune responses to influenza vaccines. PLoS One.

[21] Hilgers LA, Blom AG (2006). Sucrose fatty acid sulphate esters as novel vaccine adjuvant. Vaccine.

[22] Mahdi Abdel Hamid M, Remarque EJ, van Duivenvoorde LM, van der Werff N, Walraven V, Faber BW, Kocken CH, Thomas AW (2011). Vaccination with Plasmodium knowlesi AMA1 formulated in the novel adjuvant co-vaccine HT protects against blood-stage challenge in rhesus macaques. PLoS One.

[23] Kusi KA, Remarque EJ, Riasat V, Walraven V, Thomas AW, Faber BW, Kocken CH (2011). Safety and immunogenicity of multi-antigen AMA1-based vaccines formulated with CoVaccine HT and Montanide ISA 51 in rhesus macaques. Malar J.

[24] Faber BW, Remarque EJ, Kocken CH, Cheront P, Cingolani D, Xhonneux F, Jurado M, Haumont M, Jepsen S, Leroy O, Thomas AW (2008). Production, quality control, stability and pharmacotoxicity of cGMP-produced Plasmodium falciparum AMA1 FVO strain ectodomain expressed in Pichia pastoris. Vaccine.

[25] Faber BW, Remarque EJ, Morgan WD, Kocken CH, Holder AA, Thomas AW (2007). Malaria vaccine-related benefits of a single protein comprising Plasmodium falciparum apical membrane antigen 1 domains I and II fused to a modified form of the 19-kilodalton C-terminal fragment of merozoite surface protein 1. Infect Immun.

[26] Sirima SB, Durier C, Kara L, Houard S, Gansane A, Loulergue P, Bahuaud M, Benhamouda N, Nebie I, Faber B (2017). Safety and immunogenicity of a recombinant Plasmodium falciparum AMA1-DiCo malaria vaccine adjuvanted with GLA-SE or Alhydrogel(R) in European and African adults: a phase 1a/1b, randomized, double-blind multi-Centre trial. Vaccine.

[27] Blom AG, Hilgers LA (2004). Sucrose fatty acid sulphate esters as novel vaccine adjuvants: effect of the chemical composition. Vaccine.

[28] Roestenberg M, Remarque E, de Jonge E, Hermsen R, Blythman H, Leroy O, Imoukhuede E, Jepsen S, Ofori-Anyinam O, Faber B (2008). Safety and immunogenicity of a recombinant Plasmodium falciparum AMA1 malaria vaccine adjuvanted with Alhydrogel, Montanide ISA 720 or AS02. PLoS One.

[29] Younis SY, Barnier-Quer C, Heuking S, Sommandas V, Brunner L, Vd Werff N, Dubois P, Friede M, Kocken C, Collin N, Remarque E (2018). Down selecting adjuvanted vaccine formulations: a comparative method for harmonized evaluation. BMC Immunol.

[30] Dent AE, Bergmann-Leitner ES, Wilson DW, Tisch DJ, Kimmel R, Vulule J, Sumba PO, Beeson JG, Angov E, Moormann AM, Kazura JW (2008). Antibody-mediated growth inhibition of Plasmodium falciparum : relationship to age and protection from parasitemia in Kenyan children and adults. PLoS One.

[31] Makler MT, Hinrichs DJ (1993). Measurement of the lactate dehydrogenase activity of Plasmodium falciparum as an assessment of parasitemia. Am J Trop Med Hyg.

[32] De SL, Stanisic DI, Rivera F, Batzloff MR, Engwerda C, Good MF (2016). Plasmodium berghei bio-burden correlates with parasite lactate dehydrogenase: application to murine Plasmodium diagnostics. Malar J.

[33] Kusi KA, Faber BW, van der Eijk M, Thomas AW, Kocken CH, Remarque EJ (2011). Immunization with different PfAMA1 alleles in sequence induces clonal imprint humoral responses that are similar to responses induced by the same alleles as a vaccine cocktail in rabbits. Malar J.

[34] Chinappi M, Via A, Marcatili P, Tramontano A (2010). On the mechanism of chloroquine resistance in Plasmodium falciparum. PLoS One.

[35] Lin JT, Juliano JJ, Wongsrichanalai C (2010). Drug-resistant malaria: the era of ACT. Curr Infect Dis Rep.

[36] Muller O, Tozan Y, Becher H (2015). RTS,S/AS01 malaria vaccine and child mortality. Lancet.

[37] Heppner DG, Kester KE, Ockenhouse CF, Tornieporth N, Ofori O, Lyon JA, Stewart VA, Dubois P, Lanar DE, Krzych U (2005). Towards an RTS,S-based, multi-stage, multi-antigen vaccine against falciparum malaria: progress at the Walter Reed Army Institute of Research. Vaccine.

[38] Kusi KA, Faber BW, Thomas AW, Remarque EJ (2009). Humoral immune response to mixed PfAMA1 alleles; multivalent PfAMA1 vaccines induce broad specificity. PLoS One.

[39] Boes A, Spiegel H, Edgue G, Kapelski S, Scheuermayer M, Fendel R, Remarque E, Altmann F, Maresch D, Reimann A (2015). Detailed functional characterization of glycosylated and nonglycosylated variants of malaria vaccine candidate PfAMA1 produced in Nicotiana benthamiana and analysis of growth inhibitory responses in rabbits. Plant Biotechnol J.

[40] Miura K, Zhou H, Moretz SE, Diouf A, Thera MA, Dolo A, Doumbo O, Malkin E, Diemert D, Miller LH (2008). Comparison of biological activity of human anti-apical membrane antigen-1 antibodies induced by natural infection and vaccination. J Immunol.

[41] Rts SCTP (2015). Efficacy and safety of RTS,S/AS01 malaria vaccine with or without a booster dose in infants and children in Africa: final results of a phase 3, individually randomised, controlled trial. Lancet.

[42] Hilgers LAT, Platenburg P, Bajramovic J, Veth J, Sauerwein R, Roeffen W, Pohl M, van Amerongen G, Stittelaar KJ, van den Bosch JF (2017). Carbohydrate fatty acid monosulphate esters are safe and effective adjuvants for humoral responses. Vaccine.

